# A *Phytophthora capsici* effector suppresses plant immunity via interaction with EDS1

**DOI:** 10.1111/mpp.12912

**Published:** 2020-01-29

**Authors:** Qi Li, Ji Wang, Tian Bai, Ming Zhang, Yuling Jia, Danyu Shen, Meixiang Zhang, Daolong Dou

**Affiliations:** ^1^ Department of Plant Pathology Nanjing Agricultural University Nanjing China; ^2^ Institute of Botany Jiangsu Province and Chinese Academy of Sciences Nanjing China

**Keywords:** EDS1, PAD4, PcAvh103, *Phytophthora*, plant immunity, RxLR effector

## Abstract

EDS1 (Enhanced Disease Susceptibility 1) plays a crucial role in both effector‐triggered immunity activation and plant basal defence. However, whether pathogen effectors can target EDS1 or an EDS1‐related pathway to manipulate immunity is rarely reported. In this study, we identified a *Phytophthora capsici* Avirulence Homolog (Avh) RxLR (Arg‐any amino acid‐Leu‐Arg) effector PcAvh103 that interacts with EDS1. We demonstrated that PcAvh103 can facilitate *P. capsici* infection and is required for pathogen virulence. Furthermore, genetic evidence showed that PcAvh103 contributes to virulence through targeting EDS1. Finally, PcAvh103 specifically interacts with the lipase domain of EDS1 and can promote the disassociation of EDS1–PAD4 (Phytoalexin Deficient 4) complex in planta. Together, our results revealed that the *P. capsici* RxLR effector PcAvh103 targets host EDS1 to suppress plant immunity, probably through disrupting the EDS1–PAD4 immune signalling pathway.

## Graphical abstract

A *Phytophthora capsici* RxLR effector PcAvh103 targets host EDS1 to suppress plant immunity, probably through disrupting the EDS1–PAD4 immune signalling pathway.

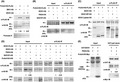

## 1 INTRODUCTION

Oomycetes are a lineage of eukaryotic microorganisms phylogenetically related to diatoms and brown algae in the kingdom Stramenopila (Jiang and Tyler, 2012). They can cause devastating plant diseases, leading to enormous environmental damage and significant economic losses worldwide (Birch *et al.*, 2006; Kamoun *et al.*, 2015). Among them, the genus *Phytophthora* is the most notorious. For example, *Phytophthora infestans* caused potato late blight and the Great Irish Famine in history (Haas *et al.*, 2009). *P. sojae* causes soybean root and stem rot, resulting in serious yield losses every year (Tyler, 2007). *P. capsici*, which infects a large number of agriculturally important vegetables like pepper, tomato, cucurbits, and eggplant, causes huge economic losses (Lamour *et al.*, 2012b). In addition, *P. capsici* can also infect the model plants *Nicotiana benthamiana* and *Arabidopsis thaliana,* therefore it has been studied as an emerging model pathogen in plant–microbe interactions (Lamour *et al.*, 2012b; Wang *et al.*, 2013). However, there are still plenty of deficiencies in understanding the infection process and pathogenic mechanism of *Phytophthora* pathogens.

During infection, *Phytophthora* pathogens secrete both apoplastic and cytoplasmic effectors to target different compartments or pathways in their hosts (Birch *et al.*, 2006; Kamoun, 2006). Among them, RxLR effectors are one class of the cytoplasmic effectors, named by their conserved Arg‐any amino acid‐Leu‐Arg (RxLR) motif at the N‐terminus (Tyler *et al.*, 2006; Jiang *et al.*, 2008). The RxLR motif was reported to facilitate delivery and translocation of effectors to host cells (Whisson *et al.*, 2007; Dou *et al.*, 2008). So far, some RxLR effectors have been reported to manipulate various aspects of plant defence (Anderson *et al.*, 2015). Recently, *P. infestans* RxLR effector SFI3 (Suppressor of early Flg22‐induced Immune response 3) was shown to target the *Solanum tuberosum* U‐box‐kinase protein (StUBK) and suppress early transcriptional responses of the pattern‐triggered immunity (PTI) pathway (He *et al.*, 2019). *P. sojae* Avh52 (Avirulence Homolog 52) recruits the cytoplasmic *Glycine max* transacetylase protein 1 (GmTAP1) into nuclear speckles, which acetylates histones H2A and H3, thereby enhancing plant susceptibility (Li *et al*., 2018). In addition, the *P. capsici* Avirulence (Avr) RxLR effector PcAvr3a12 can target and inhibit the FK506‐binding protein FKBP15‐2, which is required for endoplasmic reticulum (ER) stress‐mediated plant immunity (Fan *et al.*, 2018). Furthermore, the *P. sojae* RxLR effector Avh238 can suppress ethylene biosynthesis and facilitate infection by destabilizing soybean 1‐aminocyclopropane‐1‐carboxylate synthase GmACSs (Yang *et al.*, 2019).

To withstand infection of microbial pathogens, plants have evolved two layers of immune system. One is PTI, which uses pattern recognition receptors (PRRs) to perceive the conserved pathogen signatures called microbe‐associated molecular patterns (MAMPs), and the other is effector‐triggered immunity (ETI), which is activated through the recognition of effectors by nucleotide binding‐leucine rich repeat receptors (NB‐LRRs) (Jones and Dangl, 2006). PTI provides hosts with basal resistance to broad‐spectrum pathogens, including a reactive oxygen species (ROS) burst, mitogen‐activated protein kinase (MAPK) cascades phosphorylation, and callose deposition (Segonzac and Zipfel, 2011). Nevertheless, ETI brings about a robust defence response against specific pathogens, usually resulting in a hypersensitive response (HR) at the infection sites, which is also called programmed cell death (PCD) (Cui *et al.*, 2015).

EDS1 (Enhanced Disease Susceptibility 1) was first reported as mutations in *enhanced disease susceptibility1* (*eds1*) impair SA (salicylic acid) levels and thereby enhance susceptibility to pathogen infection (Falk *et al.*, 1999). Subsequent studies demonstrated that both nuclear and cytoplasmic EDS1 coordinate immune responses, and nuclear EDS1 is required for reprogramming of defence gene expression and basal resistance (Garcia *et al.*, 2010). EDS1 can form distinct protein complexes including the homomeric association with itself as well as heteromeric complexes with Phytoalexin Deficient 4 (PAD4) and SENESCENCE‐ASSOCIATED GENE 101 (SAG101) (Feys *et al.*, 2001, 2005). In addition, PAD4 and SAG101 contact the same N‐terminal lipase domain of the EDS1 interface, and the EDS1 heterodimers respectively mediate resistance signalling (Wagner *et al.*, 2013). EDS1–PAD4 complex works in parallel with SA in basal resistance and ETI, maintaining important SA‐related resistance genes reprogramming (Cui *et al.*, 2017). More recently, the C‐terminal EDS1–PAD4 (EP) domain surface of EDS1 enforces timely reprogramming of resistance genes (Bhandari *et al.*, 2019). However, a few studies reported that pathogen effectors can target or interfere with EDS1 as a virulence strategy. Considering EDS1 is a crucial component in plant immunity, we propose that *Phytophthora* pathogens might have evolved certain effectors that target EDS1 for virulence function.

In our study, through screening *P. capsici* effectors by using EDS1 as a bait, we uncovered an Avirulence Homolog (Avh) effector PcAvh103 and confirmed the interactions by yeast two‐hybrid (Y2H) and co‐immunoprecipitation (co‐IP) assays. We found that expression of *PcAvh103* facilitates *P. capsici* infection in *N. benthamiana*, and silencing of *PcAvh103* reduces the pathogenicity of *P. capsici*. Furthermore, we proved that PcAvh103 contributes to virulence through targeting EDS1 in *Arabidopsis*. Finally, we demonstrated that PcAvh103 specifically interacts with the lipase domain of EDS1 and can disrupt the EDS1–PAD4 complex in vivo and in vitro. Together, our results reveal that the *P. capsici* RxLR effector PcAvh103 targets host EDS1 for virulence, probably through disrupting the association of EDS1 and PAD4.

## 2 RESULTS

### 2.1 PcAvh103 interacts with EDS1

**Figure 1:**
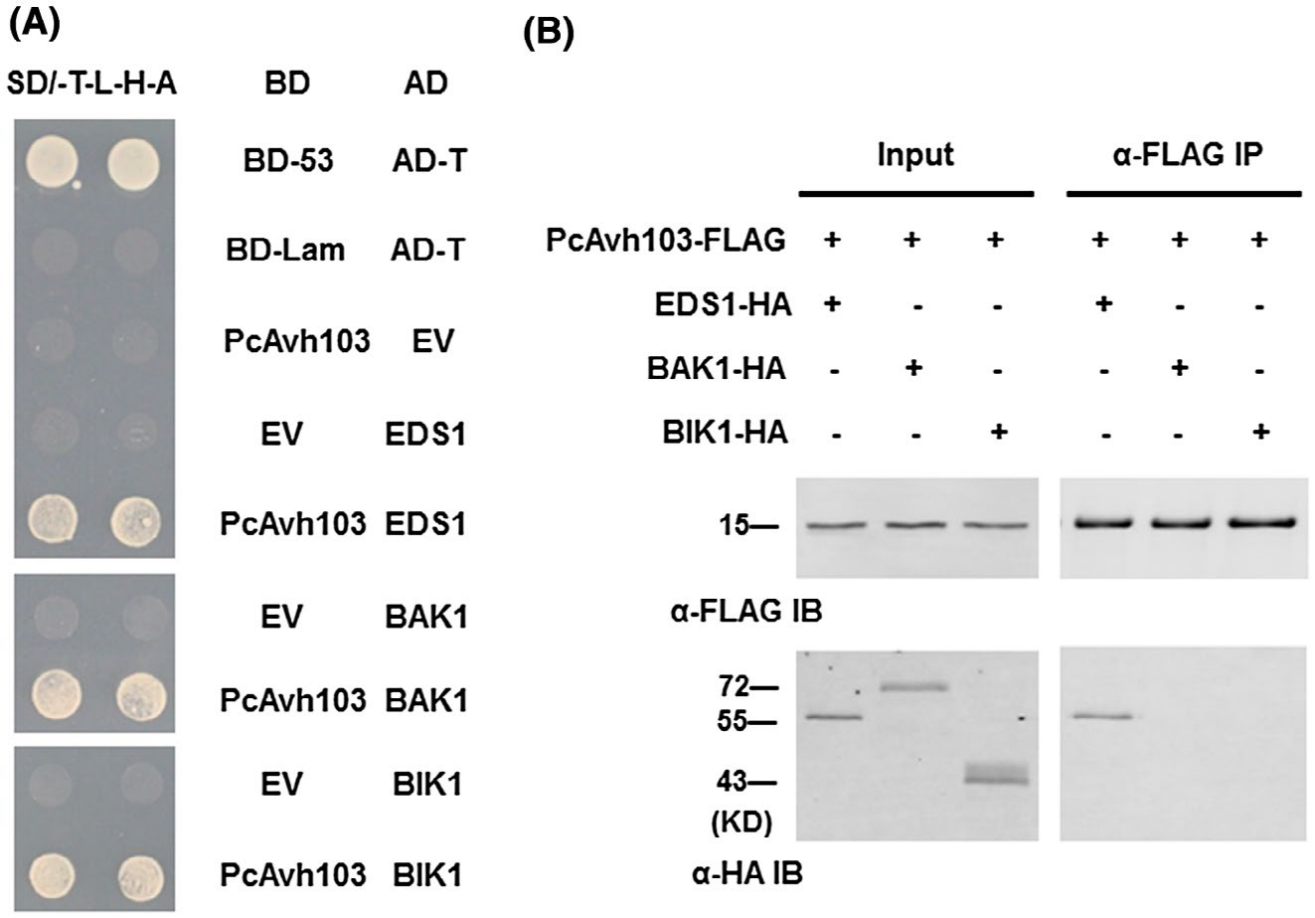
PcAvh103 interacts with EDS1. (a) Interactions between EDS1, BAK1, and BIK1 with PcAvh103 in the yeast 2‐hybrid system. Yeast AH109 cells co‐transformed with bait and prey vectors were grown on QDO (SD/−Ade/−His/−Leu/−Trp) medium. The combination of pGBKT7‐53 and pGADT7‐T was used as a positive control, while pGBKT7‐Lam and pGADT7‐T was used as a negative control. (b) Interactions between EDS1, BAK1, and BIK1 with PcAvh103 in *Arabidopsis.* Indicated constructs were transiently co‐expressed in *Arabidopsis* protoplasts. The immunoprecipitated (IP) and input proteins were analysed via immunoblot assay using anti‐FLAG and anti‐HA antibodies

EDS1 plays an important role in basal resistance and ETI‐/SA‐mediated defence response, while BAK1 (BRI1‐associated kinase 1) and BIK1 (*Botrytis*‐induced kinase 1) are core components in the PTI signalling pathway (Lin *et al.*, 2014; Cui *et al.*, 2017). To investigate whether *P. capsici* secretes effectors that target plant EDS1, BAK1, and BIK1, 42 RxLR effectors (Lamour *et al.*, 2012a) were separately cloned into prey vector and screened for interactors of EDS1, BAK1, and BIK1 using the Y2H approach (Li *et al*., 2019a, Table S1). Results showed that one effector, PcAvh103, was repeatedly identified from two independent screens (Table S1). To validate the interactions, we performed a reciprocal Y2H assay and confirmed that PcAvh103 interacted with each of the three proteins in yeast (Figure 1a). To confirm the interactions in planta, co‐IP experiments were performed by transiently expressing *PcAvh103‐FLAG*, *EDS1‐HA*, *BAK1‐HA,* and *BIK1‐HA* in *Arabidopsis* protoplasts. Only EDS1 was co‐immunoprecipitated with FLAG‐tagged PcAvh103; however, BAK1 or BIK1 were unable to bind to PcAvh103 (Figure 1b). We therefore focused on the interaction between PcAvh103 and EDS1 for further studies.

### 2.2 PcAvh103 facilitates *P. capsici* infection

**Figure 2:**
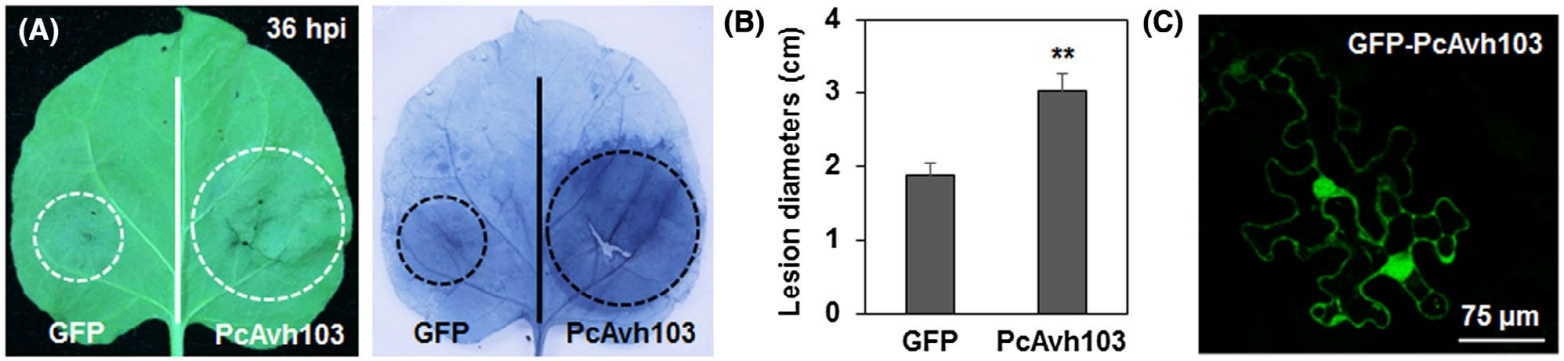
Expression of PcAvh103 can facilitate *Phytophthora capsici* infection. (a,b) *P. capsici* infection on *Nicotiana benthamiana.* Representative *N. benthamiana* leaves were inoculated by *P. capsici* LT263 after transient expression of green fluorescent protein (GFP) and PcAvh103, and photographed at 36 hr post‐inoculation. Dead cells and lesions were visualized by trypan blue staining. Lesion diameters were calculated from three independent biological replicates. Error bars represent + *SD* of at least six leaves each (**p < .01, Student's *t* test). (c) Expression of PcAvh103 in planta. Subcellular localization of PcAvh103 was visualized by confocal microscopy expressing in *N. benthamiana* epidermal cells. Scale bar represents 75 μm

To explore the virulence function of PcAvh103, we transiently expressed PcAvh103 in *N. benthamiana* and inoculated with *P. capsici. N. benthamiana* leaves expressing *GFP‐PcAvh103* and *GFP* empty vector (negative control) were inoculated with *P. capsici* zoospores onto the infiltrated area 36 hr after infiltration. Infection lesion sizes were recorded for comparison at 36 hr post‐inoculation (hpi). As can be seen, expression of PcAvh103 significantly promoted *P. capsici* colonization compared to green fluorescent protein (GFP) control, with bigger lesions (Figure 2a,b). Dead cells and lesions were visualized by trypan blue staining, further confirming that PcAvh103 promotes *P. capsici* infection (Figure 2a). We confirmed the *GFP‐PcAvh103* expression by observing the green fluorescence by confocal microscopy 48 hr after infiltration (Figure 2c). In addition, we also noticed that the GFP–PcAvh103 fluorescence signal was distributed in both the nucleus and the cytoplasm (Figure 2c).

### 2.3 PcAvh103 contributes to *P. capsici* virulence

**Figure 3:**
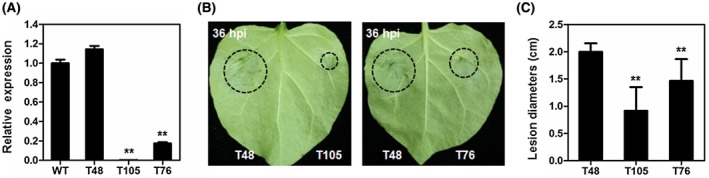
PcAvh103 is required for *Phytophthora capsici* virulence. (a) Relative transcript levels of *PcAvh103* in transformants. The transcriptional levels were determined by quantitative reverse transcription PCR with *P. capsici tubulin* gene as an internal reference (**p < .01 compared with wild‐type [WT], Dunnett's test). (b) and (c) Inoculation of *PcAvh103*‐silenced transformants. Zoospores of T48, T105, and T76 were inoculated on *Nicotiana benthamiana* leaves and photographs were taken 36 hr post‐inoculation (hpi). Lesion diameters were measured at 36 hpi with at least 12 leaves in each experiment. Asterisks indicate significant differences (**p < .01 compared with T48, Dunnett's test)

To further evaluate the contribution of PcAvh103 to the virulence of *P. capsici*, we silenced *PcAvh103* in the *P. capsici* strain LT263 (wild‐type, WT). Putative silenced transformants were selected and the silencing efficiency was estimated by quantitative reverse transcription PCR (RT‐qPCR). Two independently silenced transformants (T105 and T76) were obtained in which *PcAvh103* transcriptional levels were reduced to approximately 0% and 20% of the WT strain. An additional transformant, T48, was selected as a control in which *PcAvh103* remained unaffected (Figure 3a). To clarify whether silencing of *PcAvh103* in *P. capsici* has an effect on its growth, we checked the growth phenotype of *PcAvh103*‐silenced transformants. As shown in Figure S1, *PcAvh103*‐silenced transformants T105 and T76 exhibited similar growth rate and mycelial morphological characteristics compared to the WT and T48 strains.

The virulence of *PcAvh103*‐silenced transformants was determined on *N. benthamiana* leaves by inoculation with suspension of *P. capsici* zoospores. Compared to T48, T105 and T76 exhibited significantly reduced virulence with smaller lesions (Figure 3b). Statistical analysis showed that the lesion diameters in leaves inoculated with T105 and T76 were reduced to 46% and 69% relative to that inoculated with T48, respectively (Figure 3c). Together, these results suggest that PcAvh103 is required for *P. capsici* virulence.

### 2.4 PcAvh103 exhibits virulence function through EDS1

**Figure 4:**
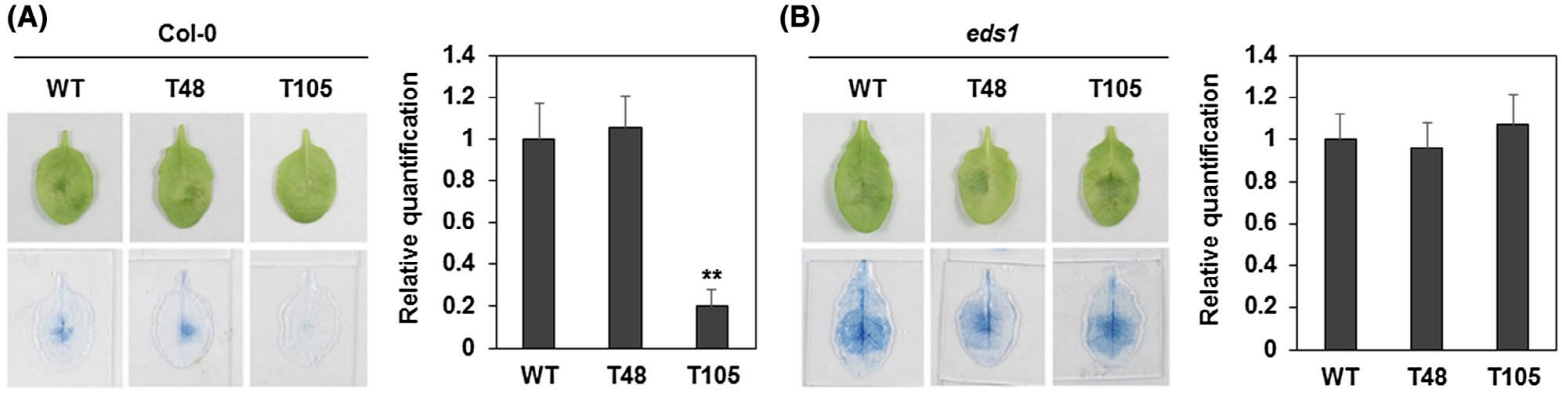
PcAvh103 contributes to *Phytophthora capsici* virulence through EDS1. (a) Inoculation of wild‐type (WT) and *PcAvh103*‐silenced *P. capsici* on *Arabidopsis*. Zoospores of LT263, T48, and T105 were inoculated on Col‐0 leaves for 36 hr. Infected leaves were stained by trypan blue. The relative biomass of *P. capsici* was measured by quantitative reverse transcription PCR. Experiments were repeated at least three times with at least 16 leaves used in each experiment (**p < .01 compared with T108, Dunnett's test). (b) Inoculation of WT and *PcAvh103*‐silenced transformants on *eds1* mutant. Details same as in (a)

Similarly, we also checked the virulence of the *PcAvh103*‐silenced transformants on *Arabidopsis* plants (Figure 4a). Leaves from Col‐0 were inoculated with zoospore suspensions of WT, T48, and T105, respectively. Compared with WT and T48 infection leaves, we noticed the reduced colonization of pathogen in T105 infection leaves, with lighter trypan blue staining observations (Figure 4a). The quantitative assay showed the pathogen accumulation in T105 infection leaves was reduced to approximately 20% of that inoculated with WT or T48 (Figure 4a). Therefore, PcAvh103 is also required for *P. capsici* infection on *Arabidopsis*.

Considering *P. capsici* is known as a soilborne pathogen, we also checked whether PcAvh103 is required for *P. capsici* infection in soil through root inoculation. We specifically implemented root inoculation of *Arabidopsis* plants with corresponding zoospore suspensions. Inoculation on Col‐0 roots by WT and T48 leads to typical disease phenotypes (leaves showing yellowing or wilting with curled leaf edges), but T105 produced notably reduced disease symptoms on Col‐0 (Figure S2). In addition, the average disease indices of Col‐0 by WT and T48 inoculation are nearly 45% and significantly higher than that of T105 (Figure S2). Together, these results demonstrate PcAvh103 is also important for root colonization.

To evaluate the consequence of PcAvh103–EDS1 association in this plant–microbe interaction, we used the T‐DNA mutant *eds1* to test the virulence of *PcAvh103*‐silenced *P. capsici* mutants. Similarly, *eds1* mutant leaves were inoculated with zoospore suspensions of WT, T48, and T105 (Figure 4b). However, no obvious difference in disease lesions was observed after inoculation treatment, and the quantitative assay also displayed a similar amount of pathogen biomass in infected leaves for WT, T48, and T105 (Figure 4b). These results indicate that PcAvh103 contributes to *P. capsici* virulence by targeting EDS1.

### 2.5 PcAvh103 has no effect on protein accumulation of EDS1

**Figure 5:**
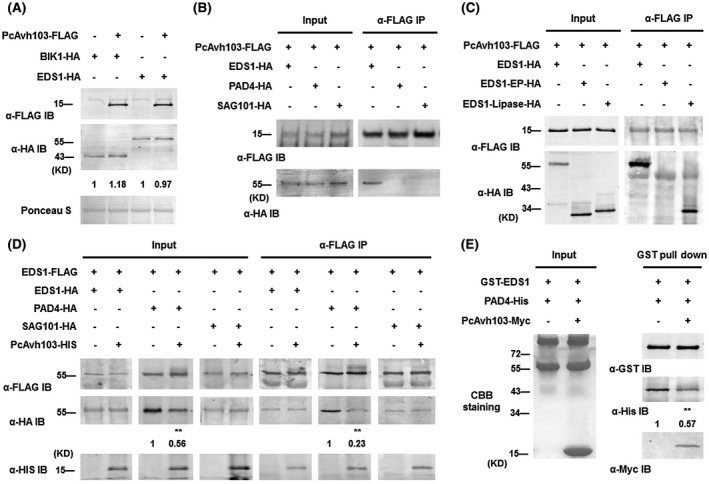
PcAvh103 can disrupt EDS1–PAD4 association. (a) Unaffected protein accumulation of EDS1 by PcAvh103. *PcAvh103‐FLAG* together with *BIK1‐HA* or *EDS1‐HA* were transiently co‐expressed in *Arabidopsis*, and protein levels were analysed by immunoblots with indicated antibodies. Numbers below represent abundances of BIK1 or EDS1 relative to solo expression. (b) and (c) Determination of the interactions in *Arabidopsis*. EDS1, PAD4 or SAG101 together with PcAvh103 (b) and EDS1, EDS1‐EP domain or EDS1‐Lipase domain with PcAvh103 (c) were co‐expressed in *Arabidopsis* protoplasts. The input and immunoprecipitated (IP) proteins were analysed via immunoblot assay using anti‐FLAG and anti‐HA antibodies. (d) Interfering with the association of EDS1 with PAD4 by PcAvh103 in vivo. Indicated constructs were co‐expressed in the presence or absence of PcAvh103 in plant cells. The immune complexes were immunoprecipitated with α‐FLAG IP, and the bound protein was detected by immunoblot with indicated antibodies. (e) Interfering with the association of EDS1 with PAD4 by PcAvh103 in vitro. Prokaryotic recombinant proteins GST‐EDS1 together with PAD4‐His were affinity purified (GST pull‐down) in the presence or absence of PcAvh103‐Myc. The gel was stained with Coomassie brilliant blue (CBB) to show equal loading of protein mixtures (input) and the amounts of bound proteins were analysed by immunoblot with indicated antibodies. Numbers below represent abundances of PAD4 relative to which in absence of PcAvh103. Asterisks indicate significant differences (**p < .01 compared with solo expression, Dunnett's test)

Interfering with the stability of crucial immune components by effectors is an effective strategy that is used by a large variety of pathogens (Li *et al*., 2019b). To test whether PcAvh103 affects the stability of EDS1 during interaction, *EDS1‐HA* or *BIK1‐HA* was transiently co‐expressed with *PcAvh103‐FLAG* in *Arabidopsis* protoplasts, and protein levels of EDS1 and BIK1 were quantified by immunoblots (Figure 5a). The results show that neither the abundance of EDS1 nor that of non‐interacted BIK1 was significantly altered by PcAvh103, compared to expression of EDS1 or BIK1 alone (Figure 5a).

### 2.6 PcAvh103 can disrupt EDS1‐PAD4 association

Previous studies reported that EDS1, PAD4, and SAG101 have homologous lipase domain and EP domain, and form the heterodimers of EDS1–PAD4 or EDS1–SAG101 at the lipase domain of the EDS1 interface, while EDS1 also strongly interacts with itself to form homomeric associations (Feys *et al.*, 2001; Wagner *et al.*, 2013). To further clarify the underlying mechanism of how PcAvh103 suppresses plant defence by targeting EDS1, we tested if PcAvh103 interferes with formation of the homomeric associations as well as in heteromeric complexes of EDS1. First, *PcAvh103‐FLAG* was co‐expressed with *EDS1‐HA*, *PAD4‐HA* or *SAG101‐HA* in protoplasts and the protein extracts were processed with co‐IP assay. The results show that PcAvh103 only interacts with EDS1 (Figure 5b). Second, we demonstrated that PcAvh103 interacts with EDS1 through its lipase domain (Figure 5c). Third, we carried out in vivo co‐IP assays between EDS1‐FLAG and EDS1‐HA, PAD4‐HA or SAG101‐HA in the presence or absence of PcAvh103‐HIS. Interestingly, expression of PcAvh103 in planta only reduced the association of EDS1 with PAD4, along with the significantly lower protein levels of PAD4 in the immunoprecipitates (Figure 5d). Finally, we performed the in vitro pull‐down assay by using GST‐EDS1 together with PAD4‐His in the presence or absence of PcAvh103‐Myc. Similarly, adding extra PcAvh103 to the system resulted in decreased enrichment of PAD4 in bound resins (Figure 5e). These findings demonstrate that PcAvh103 competes with PAD4 to bind to EDS1, thus disrupting the formation of the EDS1–PAD4 complex.

## 3 DISCUSSION

EDS1 plays a pivotal role in plant immune systems. However, few studies have reported that pathogen effectors target EDS1 or an EDS1‐related pathway to manipulate immunity. Some impressive research reported that *Pseudomonas syringae* effectors AvrRps4 and HopA1 target EDS1 and alter its interactions with RPS4/6 and SRFR1g, suggesting EDS1 might be a common virulence target that is guarded by corresponding Toll‐interleukin1‐receptor (TIR)‐NB‐LRR (nucleotide binding and leucine‐rich repeat) proteins (Bhattacharjee *et al.*, 2011). In addition, two EDS1‐like proteins in soybean (*Glycine max*), GmEDS1a and GmEDS1b, interacted with another *P. syringae* effector AvrA1 and were required for its virulence function on *rpg2* (resistance to *P. syringae* pv. *glycinea* 2) plants (Wang *et al.*, 2014). In this study, we adopted the Y2H system to screen the potential EDS1‐interacted effectors of *P. capsici*. Preliminarily, we identified an RxLR effector PcAvh103 that targets EDS1 and confirmed the interactions in yeast and *Arabidopsis*. We subsequently showed that PcAvh103 contributes to *P. capsici* virulence through EDS1 by using genetic approaches, indicating EDS1 indeed is the virulence target of PcAvh103. This is the first report about the *Phytophthora* effectors target host EDS1 for virulence, which prompted us to uncover the molecular mechanisms involved.

A variety of pathogen effectors can suppress plant immunity by using diversified strategies, termed effector‐triggered susceptibility (ETS). For example, the *P. syringae* effector HopAI1 inhibits plant MAPK cascades through a unique phosphothreonine lyase activity to suppress PTI (Zhang *et al.*, 2007). *Xanthomonas campestris* effector XopJ interferes with SA‐dependent defence response by targeting proteasomal subunit RPT6 and inhibiting proteasome activity (Ustun *et al.*, 2013). In addition, *P. sojae* effector PSR1 directly targets host PINP1, which is a previously unidentified component of RNA silencing, to promote infection (Qiao *et al.*, 2015). More recently, *P. capsici* effector RxLR207 can regulate ROS‐mediated defence response to promote the transition from the biotrophic to the necrotrophic stage by targeting *Arabidopsis* BPA1 (binding partner of ACD11) and BPLs (BPA1‐Like proteins) (Li *et al*., 2019a). In our study, we found expression of PcAvh103 significantly promotes leaf colonization of *P. capsici* and demonstrated that PcAvh103 contributes to *P. capsici* virulence through targeting EDS1 in leaves. Considering *P. capsici* is regarded as a soilborne pathogen, it is worth mentioning that PcAvh103 is also important for root colonization. Up to now, the significance of the PcAvh103–EDS1 interaction in roots has not been validated and this will be studied in the future.

Interfering with the stability of target proteins or crucial immune components is commonly used by pathogen effectors. For instance, the *P. sojae* RxLR effector PsAvh262 targets and stabilizes BiPs (binding immunoglobulin proteins) to suppress ER stress‐mediated immunity and facilitate infection (Jing *et al.*, 2016). The *Xanthomonas oryzae* pv. *oryzae* non‐TAL effector, XopK, inhibits PTI upstream of MAPK cascades by interacting with and directly ubiquitinating *Oryza sativa* somatic embryogenic receptor kinase 2 (OsSERK2), resulting in its degradation (Qin *et al.*, 2018). Another *P. syringae* type III effector, AvrPtoB, targets NPR1 (non‐expressor of pathogenesis related‐1) and mediates the degradation of NPR1 via 26S proteasome, dependent on its E3 (ubiquitin ligase) activity (Chen *et al.*, 2017). In our study, we co‐expressed PcAvh103 with EDS1 in planta, but found the protein levels of EDS1 were intact. Thus, we suggest that PcAvh103 has no effect on protein accumulation of EDS1 during interaction in *Arabidopsis*.

Previous studies reported that EDS1 can form heteromeric protein complexes with PAD4 and SAG101 (Feys *et al.*, 2005) by PAD4 and SAG101 contacting the same N‐terminal lipase domain of the EDS1 interface, to respectively mediate resistance signalling (Wagner *et al.*, 2013). In our study, we demonstrated that PcAvh103 specifically interacts with EDS1 through its lipase domain, implying that PcAvh103 may be involved in manipulating the homomeric or heteromeric immune complexes of EDS1. In vivo co‐IP and in vitro pull‐down assays revealed that PcAvh103 can only disrupt the EDS1–PAD4 association in plant cells. Hence, we speculated that PcAvh103 may target specific regions or sites in the N‐terminal lipase domain that play distinct roles in dimerization of EDS1–PAD4 or EDS1–SAG101. It is worth mentioning that an L262P exchange mutant in EDS1 lost interaction with PAD4, but not SAG101, reflecting a subtle difference between these two immune complexes (Rietz *et al.*, 2011) and thus indirectly supporting our hypothesis. Additional experiments are therefore required to test our hypothesis.

Considering the functional mechanisms of the majority of effectors are still poorly understood, we advocate that interfering with the association of immune components is also a commonly used and effective virulence strategy for pathogen effectors. For example, the *Phytophthora* effector PsAvh23 affects the formation of the ADA2–GCN5 (Alteration/Deficiency in Activation 2‐General Control Non‐depressive 5) subcomplex to manipulate host histone acetylation and reprogramme defence gene expression (Kong *et al.*, 2017). The *P. infestans* effector Pi02860 interacts with host protein NRL1 and enhances the association between NRL1 (NPH3/RPT2‐LIKE1) and SWAP70 to promote degradation of SWAP70 (He *et al.*, 2018). Furthermore, the protein accumulation level of PAD4 was lower in the presence of PcAvh103 in protein extracts as input (Figure 5c). We hypothesized disassembly of the heteromeric interactions of EDS1–PAD4 by PcAvh103 reduced the stability of PAD4, which was also reported in previous study (Feys *et al.*, 2005). Further studies are still needed to explore the interfering mechanisms used by PcAvh103.

It was reported that EDS1 and PAD4 are required for ETI response mediated by TIR‐NB‐LRR proteins, SA accumulation levels and SA‐related resistance genes responsiveness, and they also mediate basal resistance and PTI response (Falk *et al.*, 1999; Feys *et al.*, 2001). In addition, EDS1 and PAD4 are present in the nucleus and cytoplasm, and EDS1 nuclear accumulation precedes EDS1‐dependent transcriptional reprogramming (Garcia *et al.*, 2010). Loss of the association of EDS1–PAD4 compromises basal but not TIR‐NB‐LRR‐triggered resistance (Rietz *et al.*, 2011). In our study, we demonstrated that PcAvh103 can disrupt the EDS1–PAD4 association, probably contributing to suppression of EDS1–PAD4 immune signalling pathway‐mediated defence response. Hence, whether PcAvh103 can manipulate SA accumulation and responsiveness, TIR‐NB‐LRR‐triggered ETI, the nuclear–cytoplasm shuttle of EDS1, even PTI response will be explored in the future.

In summary, we identified a virulence essential effector PcAvh103 from *P. capsici*, a hemibiotrophic oomycete pathogen. PcAvh103 is required for pathogen virulence and can suppress plant defence by binding to EDS1 and disrupting the EDS1–PAD4 immune complex. This study will advance our understanding of the pathogenic mechanisms of *Phytophthora* pathogens.

## 4 EXPERIMENTAL PROCEDURES

### 4.1 Plant material and growth conditions

*N. benthamiana* plants were grown in a growth chamber at 25 °C under 16 hr light/ 8 hr dark photoperiod with a relative humidity of 60%–75%. *A. thaliana* plants were grown in a greenhouse at 23 °C with a photoperiod of 10 hr light/14 hr dark. The T‐DNA insertion mutant, *eds1* (SALK_071051) was ordered from the Nottingham Arabidopsis Stock Center (http://arabidopsis.info). The homozygous insertion lines were verified by genomic DNA PCR with primers specific for *EDS1* and the T‐DNA left border primer LB1.3 (Table S2).

### 4.2 Plasmid construction

For yeast two‐hybrid assay, *PcAvh103* was PCR‐amplified from *P. capsici* LT263 and cloned into pGBKT7, and *EDS1*, *BAK1*, and *BIK1* were amplified from *Arabidopsis* Col‐0 cDNA and cloned into pGADT7, respectively. For protoplast transfection in *Arabidopsis*, coding sequences of desired genes were amplified and cloned into the pUC19‐35S‐FLAG/HA‐RBS vector (Li *et al.*, 2005). For transient expression in *N. benthamiana*, *PcAvh103* was PCR‐amplified and inserted into pBinGFP2 vector (Song *et al.*, 2015). For prokaryotic expression of recombinant proteins, *EDS1*, *PAD4,* and *PcAvh103* were amplified and cloned into pGEX‐6P‐1, pET‐28a and pBAD/gIII, respectively. For transformation of *P. capsici*, *PcAvh103* was cloned into pHam34, which was maintained in our laboratory. Primers used for plasmids construction are listed in Table S2.

### 4.3 Yeast two‐hybrid assay

Y2H assay was performed with the Matchmaker Gold yeast two‐hybrid system (Clontech), the pGBKT7 vector was used as the bait construct, and pGADT7 as the prey construct. The bait and prey vectors were co‐transformed into the yeast strain AH109 with indicated combinations. Transformants were first selected on double synthetic dropout (DDO) medium lacking leucine and histidine, growing colonies were then plated on quadruple dropout (QDO) (SD/−Leu/−Trp/−His/−Ade) selective medium to test protein interactions. pGBKT7‐53 and pGADT7‐T co‐transformant was used as the positive control, while pGBKT7‐Lam and pGADT7‐T co‐transformant was used as the negative control.

### 4.4 Co‐immunoprecipitation assay

*Arabidopsis* mesophyll protoplasts were used for co‐IP assay and 3–4‐week‐old wild‐type *A. thaliana* (Col‐0) leaves were used for protoplasts isolation. Protoplasts isolation, PEG (polyethylene glycol)‐mediated transfection and protoplasts cultivation were performed as previously described (Li *et al.*, 2016). Protoplasts were transfected with 100 μg desired plasmids and incubated overnight. Total protein was extracted with extraction buffer (50 mM HEPES [*N*‐2‐hydroxyethylpiperazine‐*N*‐2‐ethane sulfonic acid]‐KOH [pH 7.5], 150 mM KCl, 1 mM EDTA, 0.3% Triton‐X 100, 1 mM DTT, protease inhibitor cocktail [Roche]). Protein was incubated with agarose‐conjugated anti‐FLAG antibody (Sigma) for 4 hr, washed seven times with washing buffer (50 mM HEPES [pH 7.5], 150 mM KCl, 1 mM EDTA, 0.5% Triton‐X 100, 1 mM DTT) and eluted with 3 × FLAG peptide (Sigma) for 1 hr. Immunoprecipitates were separated by SDS‐PAGE gels and detected by immunoblot using the indicated antibodies.

### 4.5 *Agrobacterium*‐mediated transient expression in *N. benthamiana*

*Agrobacterium tumefaciens* GV3101 carrying indicated vectors were cultured in Luria Bertani broth with the antibiotics kanamycin and rifampicin. Cells were harvested by centrifugation, washed three times in 10 mM MgCl_2_ and suspended in infiltration buffer (10 mM MgCl_2_, 10 mM MES pH 5.6, and 150 μM acetosyringone) to a concentration of OD_600_ = 0.5 then incubated in an incubator in dark conditions for 3 hr. The suspensions were infiltrated into fully expanded 5–6‐week‐old *N. benthamiana* leaves using a needleless syringe.

### 4.6 *Phytophthora* infection assay

The *P. capsici* (LT263) strain used in the study was maintained routinely on 10% vegetable (V8) juice medium at 25 °C in the dark. To prepare zoospores of *P. capsici*, mycelial plugs were cultured in 10% (vol/vol) V8 broth at 25 °C for 3 days and washed three times with sterilized water, then incubated in 25 °C until sporangia formed. To initiate zoospore release, the water in the plates was replaced with fresh water and incubated in 4 °C for 30 min. The zoospore concentration was adjusted by dilution in sterile water and estimated with a haemocytometer. To infect *N. benthamiana*, 10 μl zoospore suspension (approximately 500 zoospores) was drop inoculated onto the infiltration areas of a detached leaf and incubated in a growth chamber at 25 °C in darkness for 36 hr. To infect *Arabidopsis*, 5 μl droplets of zoospores (100 zoospores) were inoculated for 36 hr. Relative quantification of *P. capsici* biomass was performed to evaluate infection severity as described (Wang *et al.*, 2013). For root inoculation, *Arabidopsis* plants in pots (200 ml) were subjected to soil drench inoculation with 10 ml zoospore suspensions (10^5^ zoospores/ml). Disease development on *Arabidopsis* plants was evaluated using a disease severity index as described with disease scores ranging between 0 and 4 (Liu *et al.*, 2014). The disease index was calculated according to the formula: disease index = [(Ʃdisease grades × number of infected)/(total checked plants × 4)] × 100.

### 4.7 Confocal microscopy

A GFP‐fused construct of *RxLR103* was transformed into *A. tumefaciens* GV3101. The transient expression method on *N. benthamiana* was described above. Images were taken in a confocal laser scanning microscope (LSM 710 META, Zeiss), with an excitation wavelength of 488 nm and a 525 nm bandpass emission filter.

### 4.8 Transformation of *P. capsici*

For *P. capsici* transformation, PEG‐mediated protoplast transformation was performed as described previously (Safdar *et al.*, 2017). Putative transformants were selected on 10% V8 medium containing 30 μg/ml G418. For screening silenced transformants of *PcAvh103*, total RNA was extracted from mycelia and RT‐qPCR was conducted to characterize the silencing efficiency.

### 4.9 RNA isolation, cDNA synthesis, and RT‐qPCR

Total RNA was extracted using the RNA‐simple Total RNA Kit (Tiangen) according to the manufacturer's instructions. cDNA was synthesized using Prime Script Reverse Transcriptase (Takara). RT‐qPCR was performed using SYBR Prime‐Script RT‐PCR Kit (TaKaRa) with three technical replicates and implemented on the ABI Prism 7,500 Fast Real‐Time PCR System (Applied Biosystems Inc.). Data were analysed using the 2^−ΔΔ^ *^C^* ^t^ method.

### 4.10 Trypan blue staining assay

Inoculated leaves were stained through boiling in lactophenol–trypan blue solution (10 ml lactic acid, 10 ml glycerol, 10 g phenol, 10 mg trypan blue, all dissolved in 10 ml distilled water) for 5 min. They were then destained in chloral hydrate solution (2.5 g/ml) for 12 hr with gentle shaking. Samples were photographed under natural light.

### 4.11 Prokaryotic expression and pull‐down assay

The recombinant proteins fused with different tags were isolated from *Escherichia coli* and affinity purified following the manufacturer's instructions. For glutathione S‐transferase (GST) pull‐down assay, 5 μg GST‐EDS1, PAD4‐His, and PcAvh103‐Myc (optional) were incubated at 4 °C with 30 μl glutathione agarose beads (GE Healthcare) in a buffer containing 25 mM Tris–HCl (pH 7.5), 100 mM NaCl, and 1 mM DTT for 2 hr. The bound resins were washed five times with the incubation buffer containing 0.1% Trition‐X 100. The bound proteins were eluted with 15 mM glutathione and detected by immunoblots with indicated antibodies.

## CONFLICT OF INTEREST

The authors declare that no competing interests exist.

## AUTHOR CONTRIBUTIONS

Q.L., M.Z., and D.D. conceived and designed the research. Q.L., J.W., T.B., M.Z., and Y.J. performed the experiments. Q.L., D.S., M.Z., and D.D. analysed the data. Q.L., J.W., and D.D. wrote the manuscript.

## Supporting information


**FIGURE S1** Growth of *PcAvh103*‐silenced transformants is similar to that of WT and control strains. Photographs were taken after 3 days of culture on the 10% (vol/vol) V8 juice medium (left panel). The colony diameters were recorded and calculated (right panel). Error bars represent +*SD* of at least six plates each

**FIGURE S2** PcAvh103 is important for root colonization of *Phytophthora capsici*. Root inoculation was implemented on *Arabidopsis *wild‐type Col‐0 with zoospores suspensions of LT263, T48, and T105. Disease symptoms were photographed at 7 days post‐inoculation (left panel) and the disease indices were calculated from three independent biological replicates using at least 15 plants each (right panel). The values are means + *SEM* (**, *p* < .01 compared with wild‐type, Dunnett’s test)

**TABLE S1** Screening of RxLR effectors in *Phytophthora capsici*

**TABLE S2** Primers used in this study

## Data Availability

The data that support the findings of this study are available from the corresponding author upon reasonable request.
