# Supplementary material for: A Phytophthora capsici effector suppresses plant immunity via interaction with EDS1
Source: Mol Plant Pathol. 2020 Jan 29;21(4):502–11. doi: 10.1111/mpp.12912 (PMC7060136; doi:10.1111/mpp.12912)
Supplement: Supplementary file 1 — FIGURE S1 Growth of PcAvh103‐silenced transformants is similar to that of WT and control strains. Photographs were taken after 3 days of culture on the 10% (vol/vol) V8 juice medium (left panel). The colony diameters were recorded and calculated (right panel). Error bars represent +SD of at least six plates each [file MPP-21-502-s001.docx]

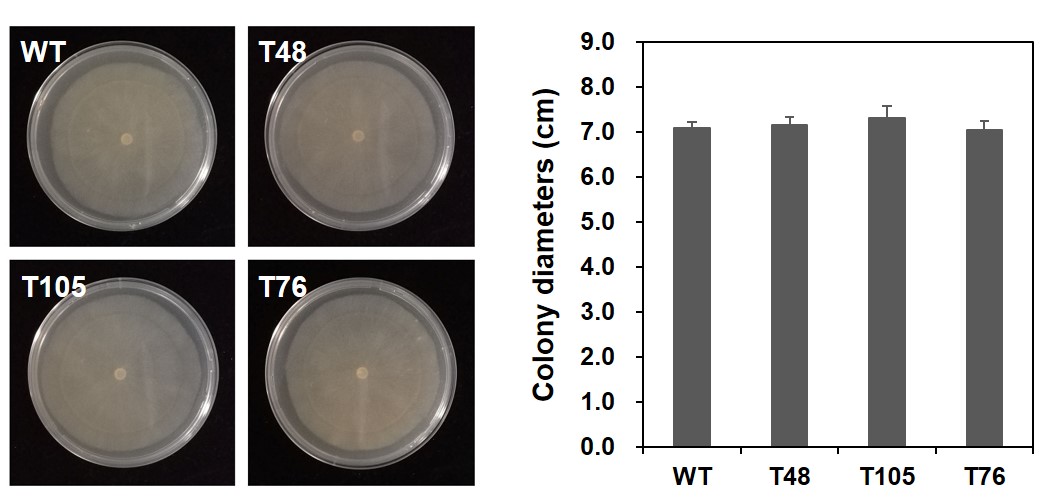


**Figure S1. Growth of *PcAvh103*-silenced transformants are similar to WT and control strains.**

Photographs were taken after 3 days of culture on the 10 % (v/v) V8 juice medium (left panel). The colony diameters were recorded and calculated (right panel). Error bars represent + SD of at least six plates each.
