# Supplementary material for: A Phytophthora capsici effector suppresses plant immunity via interaction with EDS1
Source: Mol Plant Pathol. 2020 Jan 29;21(4):502–11. doi: 10.1111/mpp.12912 (PMC7060136; doi:10.1111/mpp.12912)
Supplement: Supplementary file 2 — FIGURE S2 PcAvh103 is important for root colonization of Phytophthora capsici. Root inoculation was implemented on Arabidopsis wild‐type Col‐0 with zoospores suspensions of LT263, T48, and T105. Disease symptoms were photographed at 7 days post‐inoculation (left panel) and the disease indices were calculated from three independent biological replicates using at least 15 plants each (right panel). The values are means + SEM (**, p < .01 compared with wild‐type, Dunnett’s test) [file MPP-21-502-s002.docx]

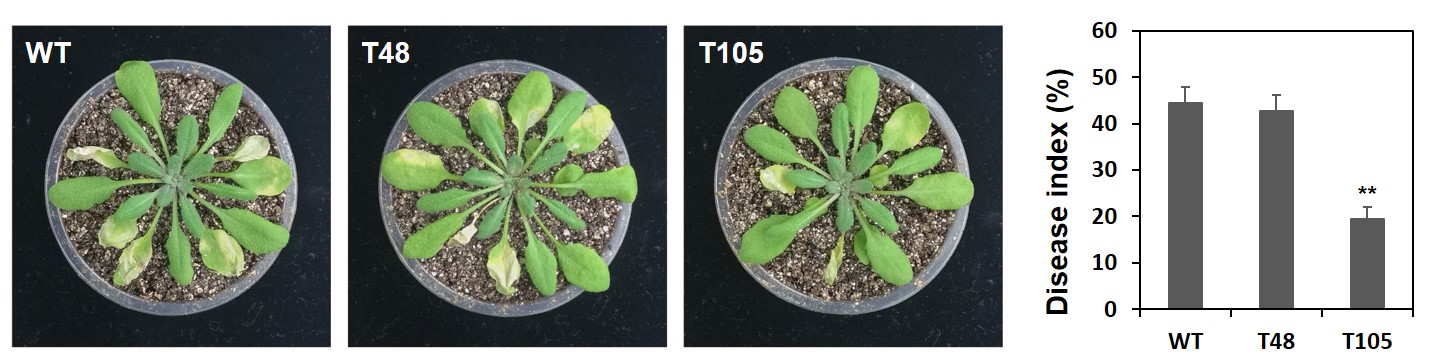


**Figure S2. PcAvh103 is important for root colonization of *P. capsici*.**

Root inoculation was implemented on *Arabidopsis* wild type Col-0 with zoospores suspensions of LT263, T48 and T105. Disease symptoms were photographed at 7 dpi (left panel) and the disease indices were calculated from three independent biological replicates using at least 15 plants each (right panel). The values are means + SEM (**, P<0.01 compared with WT, Dunnett's test).
